# A New Solution Concept for the Ultimatum Game leading to the Golden Ratio

**DOI:** 10.1038/s41598-017-05122-5

**Published:** 2017-07-17

**Authors:** Stefan Schuster

**Affiliations:** Friedrich Schiller University, Dept. of Bioinformatics, Ernst-Abbe-Platz 2, 07743 Jena, Germany

## Abstract

The Ultimatum Game is a paradigmatic two-player game. A proposer can offer a certain fraction of some valuable good. A responder can accept the offer or reject it, implying that the two players receive nothing. The only subgame-perfect Nash equilibrium is to only offer an infinitesimal amount and to accept this. However, this equilibrium is not in agreement with experimental observations, which show varying accepted offers around 40%. While some authors suggest that the fairest split of 50% vs. 50% would be explainable on theoretical grounds or by computer simulation, a few authors (including myself) have recently suggested that the Golden Ratio, about 0.618 vs. about 0.382, would be the solution, in striking agreement with observations. Here we propose a solution concept, based on an optimality approach and epistemic arguments, leading to that suggested solution. The optimality principle is explained both in an axiomatic way and by bargaining arguments, and the relation to Fibonacci numbers is outlined. Our presentation complements the Economic Harmony theory proposed by R. Suleiman and is based on infinite continued fractions. The results are likely to be important for the theory of fair salaries, justice theory and the predictive value of game theory.

## Introduction

**Figure 1:**
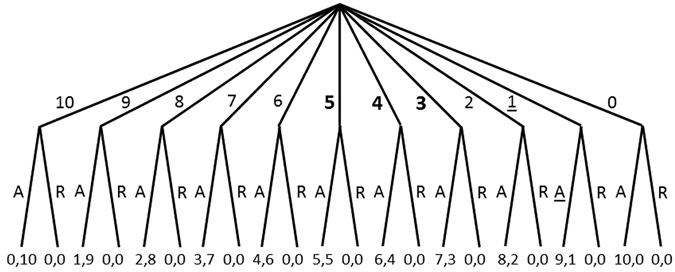
Game tree for an UG with 10 € being the total amount and 1 € being the smallest unit. The proposer chooses her strategy first and has (in this special game) 11 options. Offers are given by the numbers at the upper branches. The responder chooses second, with A and R standing for “accept” and “reject”, respectively. Payoffs are indicated below the lower branches in the sequence proposer, responder. Underlined options “1, A” correspond to the subgame-perfect Nash equilibrium. Any strategy pair “offer *p*, accept *p* and reject every lower offer” with *p* > 10% is not subgame-perfect because after the proposer had offered 1 (i.e. 10%), rejecting that would not be the best choice by the responder at that stage, that is, in the resulting subgame. Offers indicated in bold are empirically the most frequent ones and are usually accepted. The offer “4” is nearest to the GR in this special game. The offer “0” is a degenerate case because then the payoff for the responder is zero irrespective of her choice.

The Ultimatum Game is a famous asymmetric, sequential two-player game intensely studied in Game Theory. Game Theory has become a large and powerful theoretical framework, where entities such as countries^1^, companies^1^, people^1–5^, animals^1, 5^, plants^6^ and living cells^7^ down to molecules^8^ are considered as players. The Ultimatum Game (abbreviated UG henceforth) had been devised by Güth and coworkers^2^. It has the following minimalist rules: One player, called the “proposer”, is handed a valuable good, say 100 €. She is to offer any part of it to the second player, called the “responder”. The responder can choose between two strategies: to accept or to reject. If she accepts, the money is shared according to the offer. If she rejects, neither of the players receives anything. This game is both sequential and asymmetric because one player makes her move first and because the players have different sets of strategies. A decision tree for an UG with an amount of 10 € is shown in Fig. 1.

There is a slightly older bargaining game proposed by Rubinstein^9^. In that game, after every offer, the other player decides either to accept or to reject it, in which case she is to continue the bargaining. Costs of bargaining are considered, either in the form of fixed costs or of discounting factors. Note that it is not the iterated version of the UG because players swap roles after a rejection.

The standard UG is a model game for analyzing questions in economics such as fair salaries^10, 11^ and fairness in economics in general^12^. There is a case that at very large total amounts, say 10 million €, low fractional offers, say 10% might be accepted because the responder might not dare to decline 1 million €^13^. This point is still under debate in the literature. The experimental observations by Camerer and Thaler^14^ showed that the outcome changed only insignificantly if stakes were raised. Cameron^15^ performed experiments in Indonesia, which allowed her to raise stakes to three times the monthly expenditure of the average participant. The observed result was that offers were largely independent of the total amount while the responders’ acceptance rates increased at higher amounts. Andersen and coworkers^16^ reviewed the literature on this point and, by experiments in India, largely confirmed Cameron’s results. In particular, they found that sufficiently high stakes led responder behavior to converge almost perfectly to full acceptance of low offers. In what follows, we consider so-called low-stake experiments^17^, that is, we assume that the total amount is not very large (say, less than 10% of a monthly income), so that (in agreement with observations) it does not really matter for the decision, and we argue in terms of percentages.

There is a huge body of experimental results on the UG available. Roth and coworkers^18^ run the UG in four different cities: Jerusalem, Ljubljana (Slovenia), Tokyo and Pittsburgh. The results were strikingly similar. In all four locations, the modal (most frequent) offers were in the range of 0.4–0.5 and were usually accepted by the responders. Henrich and coworkers^19^ conducted a very comprehensive study with 17 different ethnic groups all over the world. The average offers ranged from 0.26–0.57 with a pronounced peak in the range 0.4–0.45 (9 ethnic groups). A meta-analysis of 37 papers with 75 results from UG experiments showed that, on average, the proposer offered 40% to the responder^20^. Offers below 0.3^14, 18, 21, 22^ or below 0.2^4, 12, 23^ are usually rejected. A clear-cut threshold is hard to determine because the considerable variance of experimental data shows that there are frequency distributions of offers and also of acceptance as a function of the offered fraction.

The game was also played with chimpanzees, using raisins as the good to be shared^24^. Responders among chimpanzees were satisfied with much lower offers (for a critical discussion, see ref. 25). These deviating results are easy to explain. Due to the lower intellectual capacities of chimpanzees in comparison to humans, they cannot anticipate the effect of rejecting low offers and focus more on the instantaneous benefit.

The UG has been studied so extensively in experiment that Camerer^26^ wrote: “we should declare a moratorium on creating Ultimatum Game data and shift attention toward new games and new theories. Good new theories codify precisely what fairness is …” (ref. 26, p. 115). In the present contribution, we address this question.

We denote the fraction the proposer wants to keep by *x*. Then the offer is 1 − *x*. Some authors distinguish between two parameters: *p* as the average offer (denoted here by 1 − *x*) and *q* as the minimum offer responders will accept, where clearly *q* < *p* and also *q* < 1 − *p* because responders would not reject a fraction they would ask for when being a proposer. To avoid a proliferation of symbols, we do not introduce general symbols for the payoffs here. It is obvious that the payoffs of the proposer and responder are *x* and 1 − *x*, respectively, if the offer is accepted and both are zero if not.

According to the classical theory, any strategy pair “offer 1 − *x*”, “accept 1 − *x*” corresponds to a Nash equilibrium, if the responder can convey a credible threat that she would reject any offer lower than 1 − *x* or when this is common knowledge^26^ (cf. ref. 1, p. 253). The strategy profile can be written as pairs (*x*, *f*(*x*)) for all *x* between 0 and 1, where *f*(*x*)) is a bi-valued function expressing whether *x* is accepted or not (denoted by A and R in Fig. 1). (*x*, *f*(*x*)) is a Nash equilibrium of the UG for some *x* if *f*(*x*) = “accept” and there is no *x*’ > *x* such that *f*(*x*’)) would be “accept”.

Somewhat paradoxically, the large number of Nash equilibria includes solutions in which the responder accepts (1 − *x*) but would reject some or all larger offers^1^. It is very plausible to restrict the solutions such that accepting some offer implies that all larger offers would be accepted as well. Even then, however, the number of Nash equilibria is too large to be applicable. Thus, the Nash equilibrium is a “weak” concept in the context of the UG and related bargaining games^9^. Thus, it is necessary to narrow down the solutions, in agreement with observations^1, 9, 12, 13^.

A frequently used way of restricting the set of solutions in sequential games is to only consider subgame-perfect Nash equilibria^1, 12, 27, 28^. That concept can be explained as follows. Sequential games can be written as decision trees (so-called extensive forms) (Fig. 1). A Nash equilibrium is subgame-perfect if at any node in the game tree, that equilibrium remains a Nash equilibrium of the subgame starting from that node^1^. If we assume that the smallest unit of the used currency is such that the lowest non-zero offer is 1% (or 10% as in Fig. 1), then the strategy pair “offer 1 − *x* = 1%, accept this” (10% in the case considered in Fig. 1) is the only subgame-perfect Nash equilibrium^1, 9, 29^, the so-called rational solution^3, 4^. This is because the responder does not have an incentive to decline that offer, as it is still larger than 0, and she cannot convey any threat after the proposer has made an offer. The proposer would not deviate either from that strategy because it grants her the largest amount.

The subgame-perfect Nash equilibrium may, alternatively, be explained from a population perspective^28^. In an initial population of responders where all of them decline any offer below 0.5, a subpopulation will start accepting lower and lower offers. This strategy would grant them an advantage, at least in the short-term. There are different opinions on whether or not games played in populations subject to infrequent mutations between strategies lead to subgame-perfect Nash equilibria^28, 30^. Gintis and coworkers^30^ found, for the UG, that evolutionary dynamics with recurrent perturbations do not converge to the subgame-perfect equilibrium as the perturbation rate approaches zero.

As outlined above, the manifold experiments with the UG in diverse ethnical communities show that proposers offer a sizeable portion of the good and low offers are usually rejected. Thus, in the case of the UG, classical theory is at odds with observations. One way of deriving solutions other than the subgame-perfect Nash equilibrium in the UG is to consider perturbations in the choice of strategy^30–32^. Here, we will derive an alternative solution concept without explicitly considering perturbations.

Although the UG is a one-shot game, most people assume that it would be iterated^21^. In experiments with a repeated UG, it was found that learning led to convergence to offers near even division (equipartition)^33^. It is generally assumed that people have learned rules of behaviour from iterated games that they apply also to one-shot games^14^. This may explain why very low offers are rejected. Responders tend to “punish” proposers who make low offers, hoping that they will make larger offers in the next round. Note that the proposer in the next round need not be the same person. In fact, the proposer may assume that in a future round of the game, she will be a responder and the proposer who was a responder before might retaliate. Since, usually, people in a population can communicate, it is common knowledge that selfishness would be punished and would imply a low reputation^21^.

In computer simulations based on an evolutionary approach, Nowak and coworkers^21^ observed that populations of players evolve their strategies so as to converge to equipartition, provided that a sufficiently large fraction of players is informed about any one offer accepted previously. The threshold of the offer below which all responders would reject converged to about 0.4. By computer simulations using adaptive dynamics and invoking a principle of empathy, similar results were found^3, 4^. Simulations indicate that taking into account stochastic effects in that players make mistakes when judging the payoffs and strategies of others favours fairness even more^32^. All these results depend, of course, on the assumptions made in the simulations.

## More Recent Developments

In the last ten years, several authors started, first independently of each other, studying the Golden Ratio (GR) in the field of Justice Research and/or in relation to the UG. The GR, often denoted by *Φ*, is defined by the value (√5 + 1))/2, which equals about 1.618, and is also known as the Golden Section or Golden Mean. The GR had been defined by Euclid in his “Elements” about 300 BC. Not mentioning the UG, Guillermina Jasso^34, 35^ wrote that the GR has come to light in the context of the justice evaluation function (JEF), which compares the actual reward and the just reward. In that context, it is worth noting that Rubinstein^9^ distinguishes the positive question, “What is the agreement reached in practice” from the normative question, “What is the just agreement”? Jasso^35, 36^ discusses the potential role of the GR in loss aversion. These ideas entered a monograph by Vermunt^37^. He stated, without proof, that a fair division (the “justice zone”) should lie between the equal split and the inverse GR, which is about 0.618. This was, in turn, discussed by Jasso^36^. She wrote: “it is possible that further theoretical work could yield a prediction for the golden ratio justice zone” (ref. 36, p. 243).

Manfred Langen suggested that the GR would be a plausible solution to the UG and published it in online magazines on economics^10, 11, 38^. Without using mathematics, he argued that a ratio that occurs frequently in nature and is known to mankind since ancient times, is accepted most easily as well-proportionate and justified.

Ramzi Suleiman^23, 29, 39, 40^ suggested the same ratio to be the solution. Suleiman had presented the idea in lectures in several talks back in 2013, e.g. at a conference in Israel^41^. His idea is based on one out of several definitions for the GR:

1 $$(1-x)/x=x.$$

**Figure 2:**
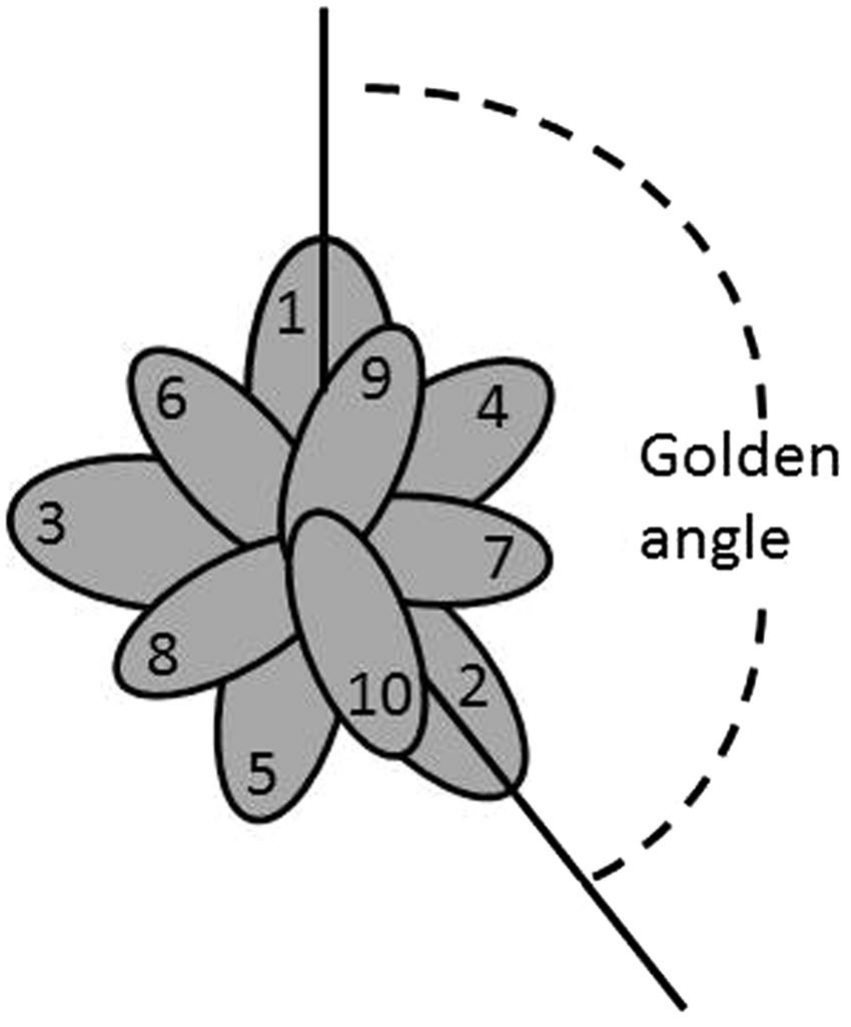
Schematic representation of plant leaves positioned according to the Golden Ratio, seen from above. The two solid lines make the Golden angle of 360°(1 − 1/*Φ*) $\cong \,137,5^\circ$. This arrangement implies that the overlap between leaves (numbered in ascending order) is minimized, since the GR can be approximated poorly by rational numbers *t*/*n*, where *t* and *n* are the number of turns and leaves, respectively. Analogously, a division of the “cake” in the UG following the GR can only be approximated poorly by ratios of small integers.

That equation says that the ratio between the smaller and larger fractions is the same as the ratio between the larger fraction and the whole. This leads to the solution

2 $${x}^{\ast }=\frac{\sqrt{5}-1}{2}\cong 0.618\ldots ,$$

which is the reciprocal of the GR. Note that x* is an irrational number. The GR is used frequently in architecture, painting and photography and occurs in phyllotaxis (leaf positions on plants)^42, 43^. The evolution of plants has led to situations avoiding that leaves would be exactly above each other, that is, that integer numbers of leaves would fit into integer numbers of turns (Fig. 2), so that they minimize taking away sunlight from the others.

The double meaning of the word “fair”, notably beautiful and equitable (just)^40^ is worth mentioning. The GR or rational numbers close to it are in striking agreement with empirical data from the UG (see above and subsection “Validation by empirical data” below).

Suleiman termed his theory Economic Harmony^23, 29, 39, 40^. The idea is that rational players strive to maximize utilities, which are functions of their actual payoffs relative to their aspired payoffs. Responders tend to accept an offer of the minor fraction of the GR, 1 − x*, because they feel that this fraction corresponds, in comparison to the larger fraction obtained by the proposer, to the ratio of the larger fraction to the whole amount. The latter is the proposer’s aspired payoff, that is, the maximum amount the proposer could get in principle. This equality of fractions can be felt by both players as a fair division.

Interestingly, when the idea underlying Eq. (1) is expressed in terms of differences rather than quotients:

3 $$x-(1-x)=1-x.$$

the solution

4 $$x=2/3$$

results (see also refs 29, 40), which is not far from the observed data either.

In contrast to Suleiman^23, 29, 39, 40^ and ideas by Vermunt^37^, several earlier authors^3, 4, 21, 27^ call the 50%: 50% solution (or a division near that ratio^44^) the fair split. To distinguish the two, we will call the 50%: 50% solution “equipartition”.

Since the UG is an asymmetric game, in which the proposer has preferential entitlement of the good, it appears to be more likely that the “fair” outcome be asymmetric than symmetric. That humans assume that the proposer has some priority is supported by the observation that a majority chooses the proposer role when they are allowed to choose^22^. See ref. 23 for a comparison between the Ultimatum and symmetric games.

I had the idea of the inverse GR being the solution in ca. 2010 and discussed it with my colleague Günter Theißen. I found that ratio intuitively appealing because it is one of the few numbers between 0.5 and 1 that can be explained in a minimalist, natural way. After coming across Suleiman’s work in the Internet, I published a first outline of my ideas^45^. To the best of my knowledge, all four lines of work (Jasso, Langen, Suleiman and myself) started independently of each other.

The GR plays a role also in other games analysed in game theory. For example, in a cyclic four-player bargaining game, a mixed Nash equilibrium exists in which the probabilities of adopting certain strategies are related to the GR^46^. In a dyadic employer-employee interaction that is related to the UG^29^, in the cognitive hierarchy model of games proposed by Camerer and coworkers^47^ and in some two-player extensive form games where the payoffs are drawn at random from a given feasible set^48^, the GR results for certain parameter settings.

Besides the GR solution, an alternative solution concept for a class of games called Pareto solvable games, which includes the UG, was proposed by Capraro and coworkers^17^. It is an extension of the concept of “cooperative equilibrium”, which assumes that players form coalitions and act cooperatively as long as they consider the outcome of the game to be fair to the coalition members^49^. This leads to the prediction that, in the case of low stakes, the average offer is 25% and responders would accept this^17^.

Here the new solution concept to the UG is presented in a much more detailed way than in ref. 45, based on an axiomatic optimality principle. The explanation is partly epistemic in that I analyse the reasoning of players in the game (cf. ref. 50). On the mathematical side, I make use of continued fractions, a tool employed in several areas in mathematics^51, 52^ and physics^53–55^. The goal of the paper is to contribute to providing the theory asked for by Camerer^26^ and Jasso^36^.

We still assume that the proposer strives to maximize her payoff (taken here as equivalent to utility) but acknowledge that the responder may be willing to sacrifice some payoff, be it in the hope that the offer will be higher next time or for reasons of justice. We assume that the proposer knows this attitude of the responder and considers it in maximizing her payoff. This entails that she will not offer too little because of fear of rejection.

## Results

### The optimality principle

Any real number between 0 and 1 can be written as a (possibly infinite) continued fraction

5 $$x=\frac{1}{{a}_{1}+\frac{1}{{a}_{2}+\frac{1}{{a}_{3}+\frac{1}{\ldots }}}}$$

with positive integer numbers *a*_*i*_^51, 52^,

6 $${a}_{i}\ge 1.$$

The restriction that all *a*_*i*_ be positive is important for making the representation as unique as possible and for confining *x* to be less or equal to 1.

Importantly, the continued fraction representation is unique and involves infinitely many levels for irrational numbers. For rational numbers, for which the continued fractions are finite, implying that all *a*_*i*_ are infinitely high from some index *i* on, there is a slight ambiguity in the last terms. For example, 1/2 can alternatively be written as 1/(1 + 1). Truncations of an infinite continued fraction yield approximations, called convergents, of the respective irrational number. The lower the *a*_*i*_, the slower the approximations converge^51, 52^.

When interpreting *x* as given by Eq. (5) as the solution of a bargaining game, the coefficients *a*_*i*_ can be considered as variables rather than constants. The following property of infinite fractions is worth noting. The proposer’s payoff (utility) *x* is a monotonic decreasing function of *a*_1_. In contrast, it is a monotonic increasing function of *a*_2_ because that parameter is under the second fraction bar. Generally, *x* is a monotonic increasing (decreasing) function of all *a*_*i*_ with an even (odd) subscript. Thus, in the context of the UG, decreasing the *a*_*i*_ with an odd subscript increases the proposer’s payoff, while decreasing the *a*_*i*_ with an even subscript increases the responder’s payoff. Moreover, the proposer has to anticipate the wishes of the responder in order to avoid that the latter rejects the offer. Thus, she strives for compromise. A good compromise is found if all *a*_*i*_ are minimized:

7 $${\rm{minimize}}\,{a}_{i}\,{\rm{for}}\,{\rm{all}}\,i=1,2,3\ldots $$

Besides the compromise argument, the optimality criterion can be substantiated by two arguments: First, the obvious unique solution

8 $${a}_{i}=1\,{\rm{for}}\,{\rm{all}}\,i$$

(cf. relation (6)) represents the simplest case of choosing the coefficients altogether. Usually, the numbers *a*_*i*_ in continued fractions are integers. Interestingly, the optimality principle (1) can even be written with real numbers, provided that the side constraint (6) is taken into account. Second, it is related to the mathematical property that the lower the *a*_*i*_, the slower the approximations converge^51, 52^. It can be proved that the continued fractions do converge^51, 52, 56^. It will become clear below why slow convergence is meaningful in the context of the UG.

Equations (5) and (8) lead to the infinite continued fraction

9 $${x}^{\ast }=\frac{1}{1+\frac{1}{1+\frac{1}{1+\frac{1}{\ldots }}}}\cong \mathrm{0.618.}$$

This is the inverse of the Golden Ratio (GR, *Φ*)^42, 51, 52, 57^, as can be seen as follows. As any infinite part of the fraction is the same as the entire fraction, we can write

10 $${x}^{\ast }=\frac{1}{1+\frac{1}{1+\frac{1}{1+\frac{1}{\ldots }}}}=\frac{1}{1+{x}^{\ast }}$$

This implies ${x}^{\ast 2}+{x}^{\ast }-1=0$, which gives the solution (2).

### The bargaining perspective

Already in the first paper by Güth and coworkers^2^, the related paper by Rubinstein^9^ and many subsequent publications^12, 18, 58^, the UG has been called a bargaining game. Although the players cannot really bargain due to the ultimatum setup, the bargaining is implicit because the proposer has to anticipate the response. Thus, it is carried out in the proposer’s mind rather than by communication. Page and Nowak^4^ refer to the concept of empathy. This opens up an epistemic approach to explaining the new solution concept, in addition to the above axiomatic approach.

We here present two ways of explanation based on bargaining arguments. Güth^58^ wrote that a vague intention of the proposer is to ask for more than 50% and that the problem to be solved is how much she can demand without risking rejection. Along the same line, Rubinstein^9^ discussed a deviation *ε* from the equal split. If *ε* is sufficiently small, the responder will prefer to agree to the proposer’s deviation. Thus, a clever manipulative maneuver by the proposer is to offer 50% − *ε* in the hope or even certainty that the responder will agree to it.

Elaborating on this idea, our solution concept can be explained as follows. The proposer asks herself whether a small deviation from *x* = 0.5 would be acceptable by the responder, say *x* = 0.51. Most responders are likely to regret renouncing to take 0.49 just because of the small deviation. In other words, they will approximate 0.49 by 0.5. Now, the same reasoning can be applied to *x* = 0.52, *x* = 0.53 etc. The reasoning applies as long as *x* can still be considered as an approximation of 0.5. In contrast, *x* = 0.65, for example, could not be considered that way anymore, because it is much nearer to *x* = 2/3, so that the responder would clearly feel that the proposer gets nearly double as much as she does. Our solution concept is based on the idea that the offer should be such that the responder cannot clearly say that the proposer gets a multiple of what the responder obtains. The GR is hardest to approximate by any rational numbers in the sense that it is furthest away from any ratio of two small integers. On the basis of that definition of “irrationality”, it can in fact be proved that the GR is the most irrational of all numbers^56^. This is related to the above-mentioned observation that the lower the coefficients in the continued fraction (5), the slower the approximations converge^51, 52^. For each convergent, there is no fraction with a smaller denominator that approximates the GR better than the fraction under consideration. The infinite continued fraction (9) converges as slowly as possible because it only contains the lowest positive integer, unity, so that the partial denominators are small^52^. This is expressed by the minimization criterion (7) subject to (6). The property of being most irrational can be understood by inspecting the optimal positions of plant leaves (Fig. 2).

**Figure 3:**
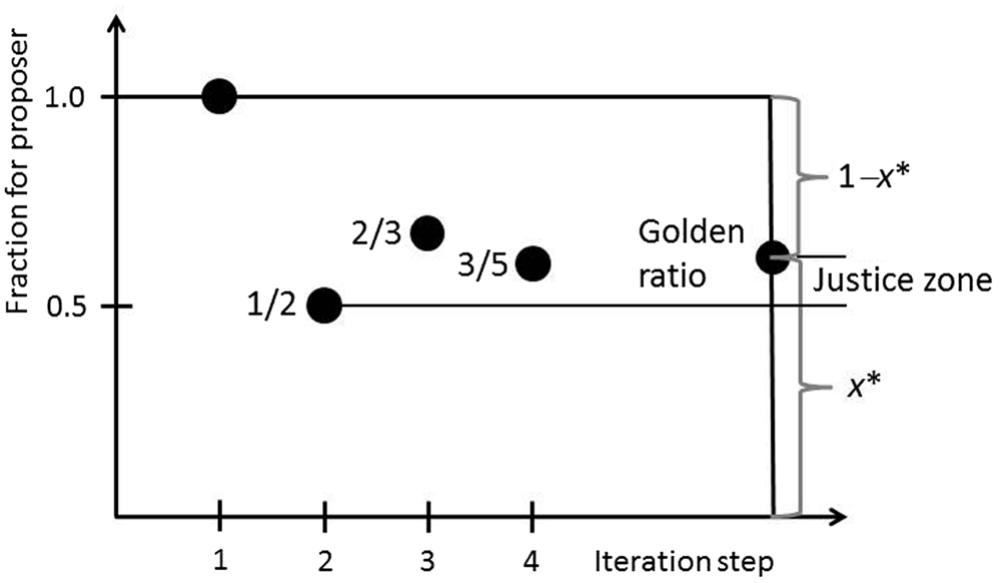
Scheme illustrating the convergence of continued fractions to the inverse Golden Ratio (0.618…). The 5^th^ convergent is 5/8 = 0.625 (not shown), which is already very near the inverse GR. The justice zone suggested by Vermunt^37^ and Jasso^36^ to be between equipartition and the inverse GR is indicated.

A second way of explanation in terms of bargaining corresponds very well with the convergents of the continued fraction (9). These are quotients of consecutive Fibonacci numbers^51, 52, 56^. A graphic representation is shown in Fig. 3. The first convergents are

11ab $${x}_{1}=1/1,{x}_{2}=1/2,$$

12 $${x}_{3}=\frac{1}{1+\frac{1}{1+1}}=\frac{2}{3}.$$

13 $${x}_{4}=\frac{1}{1+\frac{1}{1+\frac{1}{1+1}}}=\frac{3}{5}.$$

The Fibonacci numbers are defined by the recursive equation^57^

14 $${f}_{n+1}={f}_{n}+{f}_{n-1}$$

and the initial conditions

15 $${f}_{1}={f}_{2}=1.$$

This leads to the series 1, 1, 2, 3, 5, 8, 13, …. It can easily be shown that the ratio of two consecutive Fibonacci numbers tends to the GR^57^:

16 $${{\rm{l}}{\rm{i}}{\rm{m}}}_{n\to {\rm{\infty }}}\frac{{f}_{n}}{{f}_{n-1}}=\Phi =\frac{1}{{x}^{\ast }}.$$

In striking accordance with these properties of the convergents, the reasoning by many proposers can be subdivided into the following steps, thus representing an iterative bargaining.

(i) The very first estimate made by a proposer who wants to maximize her payoff without any empathy for the responder’s wishes and reasoning is to request *x*_1_ = 1, that is, to offer 1 − *x*_1_ = 0. However, the answer by the responder is clear: She would certainly decline. That first estimate is equal to the first convergent (11a) of the continued fraction (9). It can be derived from a minimization principle applied to fraction (5):

17 $${\rm{minimize}}\,{a}_{1}\,{\rm{with}}\,{\rm{all}}\,{\rm{other}}\,{a}_{i}\,(i > 1)\,{\rm{being}}\,{\rm{infinity}}$$

(ii) A very plausible next estimate comes from an empathic anticipation of the responder’s wishes and a balancing with the proposer’s wishes. The proposer asks herself: How would the responder react if I offered 1 − *x*_2_ = 0.5? She is very likely to accept because this is the fairest division. This assumption is very plausible and is used by many authors in the field.

By looking at the (not sub-game perfect) Nash equilibria with *x* < 0.5, we see that acceptance of the offer of 0.5 is not absolutely sure^13^. The responder could push up offers by rejecting offers that are equal to, or even higher than 0.5. Interestingly, the ethnical group of the Lamalera in Indonesia were found to make average offers larger than 0.5, notably 0.57^19^. However, the overwhelming majority of observations show that responders are not that hard-boiled to reject high offers, all the more as they do not have the means of communicating that threat in a one-shot game (unlike in iterated versions of the UG). Moreover, equipartition is the solution to many symmetric games.

The offer made in step (ii) corresponds to Eq. (11b). It can be derived from the minimization principle:

18 $${\rm{minimize}}\,{a}_{1},{a}_{2},\ldots {\rm{with}}\,{\rm{all}}\,{\rm{other}}\,{a}_{i}\,(i > k)\,{\rm{being}}\,{\rm{infinity}}$$

with *k* = 2. The two steps (i) and (ii) need not be gone in the given order. This does not, however, affect our line of reasoning.

(iii) Now a straightforward reasoning of the proposer is that she is privileged because being asked first. The question is by which factor the share of the responder can be diminished. The simplest possibility is to divide her share by two, that is, *x*_3_ = 2/3 (see Fig. 3). The rationale is that the ratio (1 − *x*_3_)/*x*_3_ = 1/2 would be that positive ratio less than unity that involves the smallest integers possible. This criterion can be formalized by the minimization principle (18) with *k* = 3.

The answer by the responder is more difficult to predict. Empirical observations show that some responders accept and some decline. There is a case for her declining because she may be disappointed to get only half of what the proposer gets (see above). It is too obvious that she has a pronounced disadvantage by this division.

Empathy goes one level further here: The proposer tries to anticipate whether the responder is thinking about the proposer’s priority because the proposer is asked first. A clever proposer will anticipate that the responder tries to push up offers, but still leaving more than half to the proposer. This is an argument for anticipating that the offer 1 − *x*_3_ = 1/3 will be declined. The fraction expressed by Eq. (12) can be explained as follows: The second term in the denominator, which corresponds to the responder, is divided by two because her part is weighted by 1/2 in this scenario.

(iv) Other quotients involving small integers are 2/4, which is equal to 1/2, and 1/4, which is less than 1/3 and, thus, too small. An offer that is somewhat larger than 1/3 and can still be written as a quotient of small integers is 2/5 (Fig. 3). Then, the fraction kept by the proposer is given by Eq. (13). In that equation, the second term in the large denominator is divided by a number less than two in order to somewhat increase the responder’s part. 2/5 is the offer that results from the fourth convergent of fraction (9) and can be derived from the minimization principle (18) with *k* = 4.

We can see that the convergents (truncated fractions) alternate around the inverse GR (Fig. 3). For example, 1 and 2/3 are larger, while 1/2 and 3/5 are lower than the inverse GR. This is related to the above-mentioned observation that the *a*_*i*_ with even and odd subscripts contribute in different ways to *x*, notably increasing or decreasing it, respectively.

There is a case for accepting the offer 1 − *x*_4_ (Eq. (13)) because the responder clearly gets more than half of what the proposer keeps. The disadvantage in comparison to the share of the proposer can be accepted by considering it to be minor. This, however, may inspire the proposer to slightly lower the offer. Extending the reasoning in terms of mental bargaining in a straightforward way, the offer ideally converges to the expression given in Eq. (9), which is the inverse GR.

It is worth noting that Fibonacci numbers and the GR occur in various problems in physics^53–55, 59^. A prominent example is provided by resistance ladders^53, 54^. In fact, these can be used for illustrated the reasoning in terms of convergents (Supplementary Information).

The GR solution also has the following interesting property (for a proof, see Supplementary Information). Assume that the proposer will play the same game with a second responder, using her part *x* and offering the same fractional part of it as she offered before to the first responder. If the proposer divides according to the GR, the proposer keeps, after the second game, as much as the first responder received in the first.

### The 60:40 approximate solution

As the proposer has to express her offer in usual numbers rather than by mentioning the GR or the square root of 5, she will usually round the offer. Due to our decimal numeral system, a straightforward way of rounding the GR is 60%, thus offering 40% to the responder. As 60% = 3/5, this is again a ratio of Fibonacci numbers (Eq. (13)). This coincidental equality of the rounded value with a ratio of Fibonacci numbers makes this approximate solution very appealing. It is in agreement with a frequently observed offer^19, 20, 40^.

Another argument for the 60:40 solution is that it is risky to offer the lowest amount she thinks the responder will accept. It certainly increases chances of acceptance if the offer is increased by a certain margin. This may lead to an offer of 40% rather than 38.1%. However, whether 40% are offered depends on the amount to be divided, that is, for example, whether 10 €, 9€, 8€ etc. are at stake. Offers are often rounded to full or half units of the respective currency rather than to full 10 percent units.

It is worth noting that there are other number series that lead to the GR as well, for example, the Lucas numbers^57^. These are less relevant for the solution concept presented because the convergents of the infinite continued fraction (9) involve Fibonacci numbers rather than any other numbers. For a more detailed discussion, see the Supplementary Information.

### Validation by empirical data

Both the “ideal” solution of the GR and the “justice zone” are, in contrast to the concept of subgame-perfect Nash equilibrium, appealing due to their excellent agreement with the empirical values from the literature (cf. Introduction). Inspecting the observations reported by Henrich and coworkers^19^ in more detail shows that the mean value of offers observed with the ethnical group of Gnau in Papua New Guinea, 0.38, is closest to the GR. Also very close are the average offers observed with the Kazakh (0.36), Hadza (0.40), and Tzimane (0.37). Although it is speculative at this point to draw a conclusion, the coincidence of the average offer of 0.40 observed with the Hadza with *x* = 3/5, the convergent shown given in Eq. (13), is worth mentioning.

Suleiman^40^ showed by a Two One-Sided Test (TOST) that the agreement of the theoretical prediction in terms of the GR is statistically significant both with the empirical data compiled by Henrich and coworkers^19^ and with the meta-analysis by Oosterbeek and coworkers^20^. For the data from the latter paper and with a confidence level of 10%, the *p*-values for the upper bound and lower bound were computed to be <0.0001 and 0.0425, respectively^40^. In the original paper by Güth and coworkers^2^, the average accepted offer in the “naïve decision behaviour in easy games” (Table 4 in ref. 2) was 37,65%, in striking agreement with the GR. Seven out of 19 accepted offers were 0.5 (included in the computation of the average). One proposer requested 5.55 DM out of 9 DM, which corresponds to an offer of 0.3833.

Performing a precise statistical test with the data on the UG is difficult because of the rather wide and complex distribution of data. Thus, it is of interest to look not only at the average values from the experimental studies but also at the distribution of offers. A considerable fraction of people indeed make the equipartition offer of 0.5^2, 19, 27^. This is well explained by the second convergent as given in Eq. (11b). Figure 2 in ref. 19 shows that 1 − *x* = 2/5 (convergent *x*_4_, Eq. (13)) was the most frequent offer in the groups of Aché and Gnau, the second-most frequent offer in the group of Shona and in Pittsburgh, while the by far most frequent offer in the Toruud group was very near to 3/8 (corresponding to convergent *x*_5_).

An observation reported by many authors is that offers below 0.3 are generally rejected^14, 18, 21, 22^. This can be explained better by the GR solution than by a 2/3 vs. 1/3 solution or equipartition.

## Discussion

Here we have presented a new solution concept for explaining the hypothesis that a division according to the Golden Ratio (GR) would characterize the relevant Nash equilibrium of the Ultimatum Game (UG). That equilibrium implies that the proposer offers about 38.2% and the responder accepts that or any higher fraction while she would reject any lower fraction. Our solution concept is based on an optimization criterion, which can be interpreted by epistemic arguments or by a bargaining perspective. To disguise the disadvantage for the responder best, it is favourable for the proposer to make an offer that is farthest away from any ratio of integer numbers, that is, an offer near the inverse GR. Such an offer is hardest to evaluate by the responder in view of whether it is still near equipartition. It is the natural boundary above which an unjustified inequity is no longer accepted^10^. In the light of the observation that people have learned rules of behaviour from iterated games that they apply also to one-shot games^14^, we here use arguments from both perspectives: that of one-shot games and that of iterated games.

Our solution concept to the UG serves to narrow down the extremely large number of Nash equilibria of the UG to one solution (the inverse GR) or an interval between 1/2 and the inverse GR, even though these solutions are not sub-game perfect. Our above arguments are alternative and complementary to the explanation given by Suleiman^39, 40^. In game theory in general, several alternative solution concepts^50^ such as correlated equilibrium^60^, Kantian equilibrium^61^, and cooperative equilibrium^49^ have been proposed, which even imply solutions that differ from Nash equilibria^50, 60, 61^. Correlated equilibrium means that in games where mixed strategies are relevant (such as in a coordination game or Hawk-dove game in the case where information exchange between players is excluded), the average payoff is increased for both players when the decision probabilities are correlated^60^. In the concept of Kantian equilibrium^61^ and the related co-action concept^62^, each agent maximizes her payoff assuming that all other agents in a symmetric situation will make the same decision. These concepts were devised for non-sequential games, so that they are not (immediately) applicable to the UG (which is, in addition, asymmetric). The key idea of the cooperative equilibrium concept^49^ is that players form coalitions (see Introduction). The average offer of 25% predicted on the basis of that concept^49^ is, however, less well in agreement with observed data than our prediction of 38.2%. So is the equilibrium offer of a little more than 20% predicted by Gale and coworkers^31^ (these authors mentioned, though, that their results depend very much on the specifications of the model).

It is worth noting that all the Fibonacci convergents to the inverse GR from 1/(1 + 1) on lie in the justice zone proposed by Vermunt^37^ and Jasso^36^. Justice theory predicts a single point of perfect justice (equipartition in the case of the UG) and a continuum of justice evaluation magnitudes^36^. Along those lines, one may argue that the set of Nash equilibria should be narrowed down to the region between 0.5 and the inverse GR rather than to one Nash equilibrium only.

The (rational or intuitive) reasoning of the proposer may start at equipartion and decrease potential offers until the lower fraction of the GR is reached, because that is the boundary of what can be approximated by 1/2. Or the proposer may follow an alternating iteration in her mind in terms of predicted upper and lower bounds of what would probably be accepted by the responder. This is usually made in terms of ratios of small integers. Persons with a strong intuition may perform that iteration almost instantaneously. In future experiments, test persons may be asked (e.g. in questionnaires) which way they followed in reaching their decision.

It is certainly of interest to investigate, in theoretical and experimental studies, iterated versions of the UG (see ref. 33 and refs cited therein). There are different possible versions of iteration: The players may keep the total payoff of all rounds, or only the payoff of the last round, the numbers of rounds may or may not be known a priori, proposers may meet new responders or always the same, players may have full or incomplete information about previous outcomes etc.

As mentioned above, the GR is relevant in phyllotaxis^42^ and can there be derived from an optimality principle as well^43^. There appears to be a relationship between that observation and our solution concept (Fig. 2). In the UG, the division is made so as to avoid that the fraction claimed by the proposer is a clear multiple of the offer.

Above, we have explained the frequently observed offers of ca. 33%^58^ and 40%^19, 20, 23^ in that many persons would stop the iteration at 1/3 or 2/5. The proposer needs to express her offer in the decimal system or a ratio of integers rather than by an intuitively felt ratio. Thus, it is unlikely that she would propose 38.2%. The offers 1/3 and 2/5 correspond to two Fibonacci approximations for the GR. And so does the equipartition solution 1/2. This is reminiscent of the various approximations of the GR by ratios of Fibonacci numbers realized in phyllotaxis^42, 43^. Ratios of two consecutive Fibonacci numbers (turns divided by number of leaves) are realized by numerous plants, apparently with lower effort than the GR. The special case of alternate leaves (standing on opposite sides) corresponds to a ratio of 1/2. It can be assumed that the less effort is spent, the less accurate is the approximation of the Golden Ratio. The Fibonacci series is also relevant in many other applications, for example, the enumeration of polyphenanthrenes in chemistry^63^ and fatty acids^64^. The GR also arises in the solution of some problems in electric and fluidic circuits^53, 54^ and nonlinear dynamics in physics^55, 59^ (see Supplementary Information) and in dynamic optimization of biochemical pathways^65^.

The alternative (or, in a sense, approximative) solution 2/3:1/3 can be explained in at least three other ways: The difference between the two shares is the same as the difference between the proposer’s share and what she could get in principle (see Eq. (3)). It is also achieved when the proposer and responder aspire to obtain the entire “cake” and half of it, respectively, and find a trade-off^40^. Another explanation follows from a bargaining perspective (Thomas Pfeiffer, personal communication): When the responder asks to get half of the money, the proposer asks to get half of that (i.e. 1/4) back, the responder asks to get 1/8 back and so forth, the geometric series converges to an offer of 1/3. The 2/3:1/3 solution also arises in one variant of the dyadic employer-employee interaction analysed by Suleiman^29^.

Although I do not claim that the above results can directly be applied to more complex bargaining situations in which one side has a priority whatsoever, such as salary negotiations, there are obvious relationships to such situations^10, 11, 29, 38^. Offered salaries can be accepted or rejected by employees. If all employees reject the offer and do not work, neither side gets anything. At the scale of societies, too low wages lead to social unrest. Langen^10, 11^ suggested (without proof) that a fair salary system in a hierarchically structured company should imply that the income increases from level to level by the Golden Ratio (cf. also refs 34–36). As mentioned by Langen^10, 11^, until two decades ago, there was an unwritten rule in large companies that the salary of a top manager should not exceed twentyfold the wage of a skilled worker. On the basis of a Golden Ratio system, this corresponds to 6–7 levels in the hierarchy. This is a realistic number in large companies and corresponds to the “magic” number of seven, that is, the number of objects an average human can hold in working memory^66^. Thus, the rule of the ratio of 20 might be based on an empirical application of the Golden Ratio.

As mentioned in the Introduction, continuous frequency distributions of offers and of the acceptance probability as a function of the offered values can be determined empirically. It will be very interesting in future theoretical studies to compute optimal frequency distributions by variational calculus. This may be related to mixed Nash equilibria, which have scarcely been analysed for the UG so far^17, 31^. A characteristic value would be that offer which is accepted with 50% probability. It may be hypothesized, based on the above line of reasoning, that this characteristic value would be near the inverse GR.

The UG might even be relevant in microbiology. Several micro-organisms show the so-called sequential cross-feeding^67, 68^. That is, a product excreted by one of them is taken up as a nutrient by the other. For example, some strain of the bacterium, *Escherichia coli* may take up glucose and convert it into acetate. This can be taken up by another strain of the same species, which converts it further into CO_2_ and water. During evolution, the two strains or species must come to an agreement about the metabolic intermediate to be excreted and taken up. It might have been ethanol or lactate (as is the case for other pairs of species) rather than acetate. If the responder rejects the offered intermediate substance, the proposer will not survive either or at least be harmed because substances such as acetate and ethanol are toxic in higher concentrations, which is very reminiscent of an UG. As the present-day intermediates such as acetate, ethanol and lactate are much poorer substrates than glucose, the observed accepted offer is less valuable than 50%. On the other hand, they are not the poorest substrates possible^69^, so that they do not correspond to the subgame-perfect equilibrium. This can be understood from a population perspective. If the responders only obtain very little energy, they cannot build up a population size sufficient to remove the harmful intermediates. Thus, proposers have an interest in enabling responders to maintain sufficiently high population densities, wich requires making sufficiently high offers (reminiscent of salary negotiations). Studies on cross-feeding pathways passing several organisms may be inspired by earlier calculations based on dynamic optimization of metabolic pathways, which indeed led to the GR^65^.

### Data availability statement

All data used are given in the paper or are taken from references that are properly cited.

## Electronic supplementary material

Supplementary doc File
