# Supplementary material for: A New Solution Concept for the Ultimatum Game leading to the Golden Ratio
Source: Sci Rep. 2017 Jul 17;7:5642. doi: 10.1038/s41598-017-05122-5 (PMC5514050; doi:10.1038/s41598-017-05122-5)
Supplement: Supplementary file 1 — Supplementary doc File [file 41598_2017_5122_MOESM1_ESM.doc]

**Supplementary Information for:**

**A New Solution Concept for the Ultimatum Game**

**leading to the Golden Ratio**

**Stefan Schuster**

Dept. of Bioinformatics, Friedrich Schiller University,

Ernst-Abbe-Platz 2, 07743 Jena, Germany

e-mail: stefan.schu@uni-jena.de

1. **An electrical analog**

The new solution concept can be illustrated by an electrical analog^53^ (Suppl. Fig. 1). A voltage applies at two points A and B. This causes an electric current that depends on the resistance between the two points. We assume that all particular resistances are equal to each other in value; they can be normalized to be unity. The different subscripts serve to distinguish their location in the circuit.


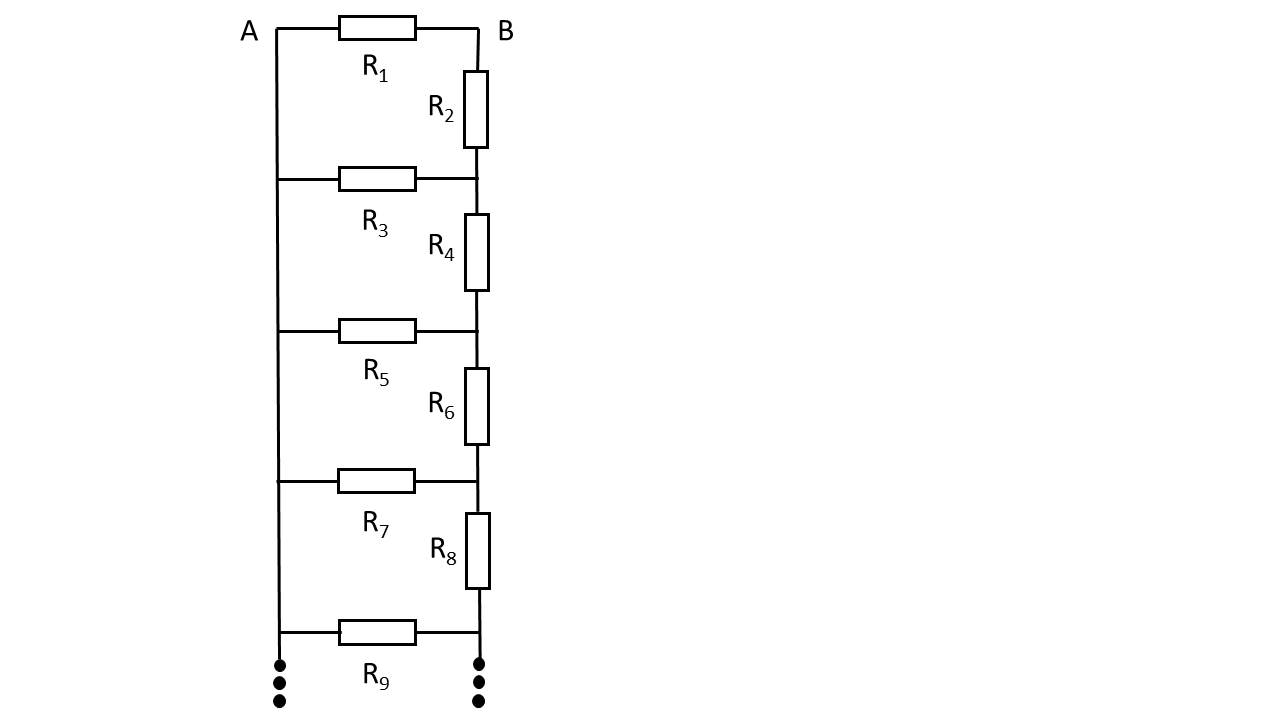


**Supplementary Figure 1.** Resistance ladder for illustrating continued fractions^53^. All resistances are equal to each other in value; the different subscripts only serve to distinguish their location. By building up the ladder in the downward direction (i.e. with increasing subscripts), the overall resistance changes according to the convergents of the continued fraction given in Eq. (9).

We now illustrate the iterative bargaining in the mind of the proposer outlined in the main text (subsection “The bargaining perspective”). The first thought of the proposer of taking all the money corresponds to a current just across resistance R_1_, which corresponds to Eq. (11a). The second thought – dividing the money evenly – corresponds to adding resistance R_2_ in parallel, which corresponds to Eq. (11b). Given that the values of R_1_ and R_2_ are equal, both currents are equal. The fact that the total current is then double as high as the current in the first case need not spoil our argument; we consider relative currents. Now a natural reasoning of the proposer is that she is privileged because being asked first. The simplest possibility is to cut the responder’s share by two. In the circuit, this can be realized by adding R_3_, leading to a relative current across the bypass of 1/3 (Eq. (12)).

A crucial point in our justification of the proposed solution is that the responder will not accept receiving 1/3 of the good only. The fact of being disadvantaged is too obvious, since the proposer gets double as much. To increase chances that the responder accepts, a logical „move“ by the responder is to somewhat increase the responder’s share. This can be done by alleviating the resistance on the responder’s side by another parallel current. As halving the entire resistance on the responder’s side would again lead to the 2:1 solution, a logical next step is to halve R_3_ only, by resistance R_4_, as shown in Suppl. Fig. 1. This leads to the 3:2 solution, since the resulting relative currents across the bypass and across R_1_ are 2/5 and 3/5, respectively, corresponding to Eq. (13). This may or may not be the endpoint of reasoning. If not, it can be continued in a straightforward way. Eventually, this leads to a highly symmetric assembly of infinitely many resistances.

It is known since the 1950’s in the theory of Fibonacci numbers that the total resistance of the assembly up to R*_n_* is the *n*-th convergent of the continued fraction (9) and that it tends to the GR^53^. Resistance ladder networks including their analogs in fluid dynamics have been studied intensely^54^.

1. **Dynamical systems**

In the main text, we outlined the reasoning that the offer in the UG should be away from some ratio of small integers as far as possible and the relevance of that principle also for phyllotaxis. The same reasoning is also relevant in some problems in dynamical systems in physics^55,59^. It is related to the so-called KAM theorem, which deals with the persistence of quasiperiodic motions under small perturbations^59^. Chaotic behaviour can be avoided best when the ratio between the frequencies of two coupled nonlinear oscillators (e.g. planets on their orbits) is as irrational as possible, that is, if it is the GR. This can again be proved by infinite continued fractions^59^.

1. **Playing the game a second time**

The GR solution has the following interesting property. Assume that the proposer will play the same game with a second responder, using her part *x* and offering the same fractional part of it as she offered before to the first responder. We here denote the second offer by *y*. Then

$\frac{1-x}{1}=\frac{y}{x}$ (S1)

We can now ask how large *y* should be such that, after the second game, the proposer keeps as much as the first responder received in the first game:

*x*−*y* = (1−*x*) (S2)

Eqs. (S1) and (S2) lead to the quadratic equation *x*^2^ + *x* − 1 = 0 and, thus, to the inverse GR.

1. **Lucas numbers**

There are other number series that lead to the GR as well, for example, the ratio of two consecutive Lucas numbers^57^. The Lucas numbers 1, 3, 4, 7, 11, … are defined by the recursion formula (14) as well, but start with

*f*_1_ = 1 , *f*_2_ = 3 . (S3a,b)

Although the convergents of Eq. (9) are Fibonacci numbers rather than Lucas numbers, let us check whether ratios of consecutive Lucas numbers could provide a convenient approximate solution to the UG. *x* = 1/3 would certainly be too small, *x* = 3/4 would probably be too high to provide a general solution, although an offer of 25 % is sometimes made and accepted^2^. 4/7 = 0.571… and 7/11 = 0.636… are candidate solutions but are much less convenient than 3/5 because the latter matches our decimal numeral system much better. In the limit, ratios of Lucas numbers tend to the inverse GR anyway; then there is no significant difference to ratios of Fibonacci numbers.
